# Development of a Bone-Mimetic 3D Printed Ti6Al4V Scaffold to Enhance Osteoblast-Derived Extracellular Vesicles’ Therapeutic Efficacy for Bone Regeneration

**DOI:** 10.3389/fbioe.2021.757220

**Published:** 2021-10-26

**Authors:** Kenny Man, Mathieu Y. Brunet, Sophie Louth, Thomas E. Robinson, Maria Fernandez-Rhodes, Soraya Williams, Angelica S. Federici, Owen G. Davies, David A. Hoey, Sophie C. Cox

**Affiliations:** ^1^ School of Chemical Engineering, University of Birmingham, Birmingham, United Kingdom; ^2^ School of Sport, Exercise and Health Sciences, Loughborough University, Loughborough, United Kingdom; ^3^ Trinity Centre for Biomedical Engineering, Trinity Biomedical Sciences Institute, Trinity College Dublin, Dublin, Ireland; ^4^ Department of Mechanical, Manufacturing and Biomedical Engineering, School of Engineering, Trinity College Dublin, Dublin, Ireland; ^5^ Advanced Materials and Bioengineering Research Centre, Trinity College Dublin and RCSI, Dublin, Ireland

**Keywords:** 3D printing, titanium, extracellular vesicles, osteogenesis, tissue engineering

## Abstract

Extracellular Vesicles (EVs) are considered promising nanoscale therapeutics for bone regeneration. To date, EVs are typically procured from cells on 2D tissue culture plastic, an artificial environment that limits cell growth and does not replicate *in situ* biochemical or biophysical conditions. This study investigated the potential of 3D printed titanium scaffolds coated with hydroxyapatite to promote the therapeutic efficacy of osteoblast-derived EVs. Ti6Al4V titanium scaffolds with different pore sizes (500 and 1000 µm) and shapes (square and triangle) were fabricated by selective laser melting. A bone-mimetic nano-needle hydroxyapatite (nnHA) coating was then applied. EVs were procured from scaffold-cultured osteoblasts over 2 weeks and vesicle concentration was determined using the CD63 ELISA. Osteogenic differentiation of human bone marrow stromal cells (hBMSCs) following treatment with primed EVs was evaluated by assessing alkaline phosphatase activity, collagen production and calcium deposition. Triangle pore scaffolds significantly increased osteoblast mineralisation (1.5-fold) when compared to square architectures (P ≤ 0.001). Interestingly, EV yield was also significantly enhanced on these higher permeability structures (P ≤ 0.001), in particular (2.2-fold) for the larger pore structures (1000 µm). Furthermore osteoblast-derived EVs isolated from triangular pore scaffolds significantly increased hBMSCs mineralisation when compared to EVs acquired from square pore scaffolds (1.7-fold) and 2D culture (2.2-fold) (P ≤ 0.001). Coating with nnHA significantly improved osteoblast mineralisation (>2.6-fold) and EV production (4.5-fold) when compared to uncoated scaffolds (P ≤ 0.001). Together, these findings demonstrate the potential of harnessing bone-mimetic culture platforms to enhance the production of pro-regenerative EVs as an acellular tool for bone repair.

## Introduction

Bone fractures caused by traumatic injury or common age-associated disorders, such as osteoporosis, present an enormous healthcare and socioeconomic burden worldwide (Baroli, 2009; Dimitriou et al., 2011), with 10 million people suffering with musculoskeletal disorders in the United Kingdom alone (Chance-Larsen et al., 2019). This is anticipated to increase further in the future, due to the growing ageing population and the demand for sustained quality of life in the older years (Amini et al., 2012). Autologous bone grafts have been seen as the gold standard clinical therapy for many years, as they are histocompatible and non-immunogenic. However, studies have reported the considerable issues associated with this treatment including donor site morbidity, limited availability and risk of infection (Swan and Goodacre, 2006; Oryan et al., 2014). Consequently, there is an urgent demand for novel therapeutic approaches for bone augmentation strategies. Engineered bone tissue has been seen as a potential alternative to conventional bone graft treatments, however, there are limitations regarding the translation of cell-based therapies to the clinical setting including their inherent heterogeneity, uncontrolled differentiation, immune rejection, functional tissue engraftment and neoplasm formation (Amariglio et al., 2009; Herberts et al., 2011). Moreover, the translation of cell-based treatments are hampered due to issues such as intensive cost, government regulations and ethical issues (Izadpanah et al., 2006; Heathman et al., 2015; Volarevic et al., 2018). Hence, there is growing precedence to develop cell-free or acellular technologies to promote bone regeneration (Burdick et al., 2013; Wang et al., 2018).

**GRAPHICAL ABSTRACT:**
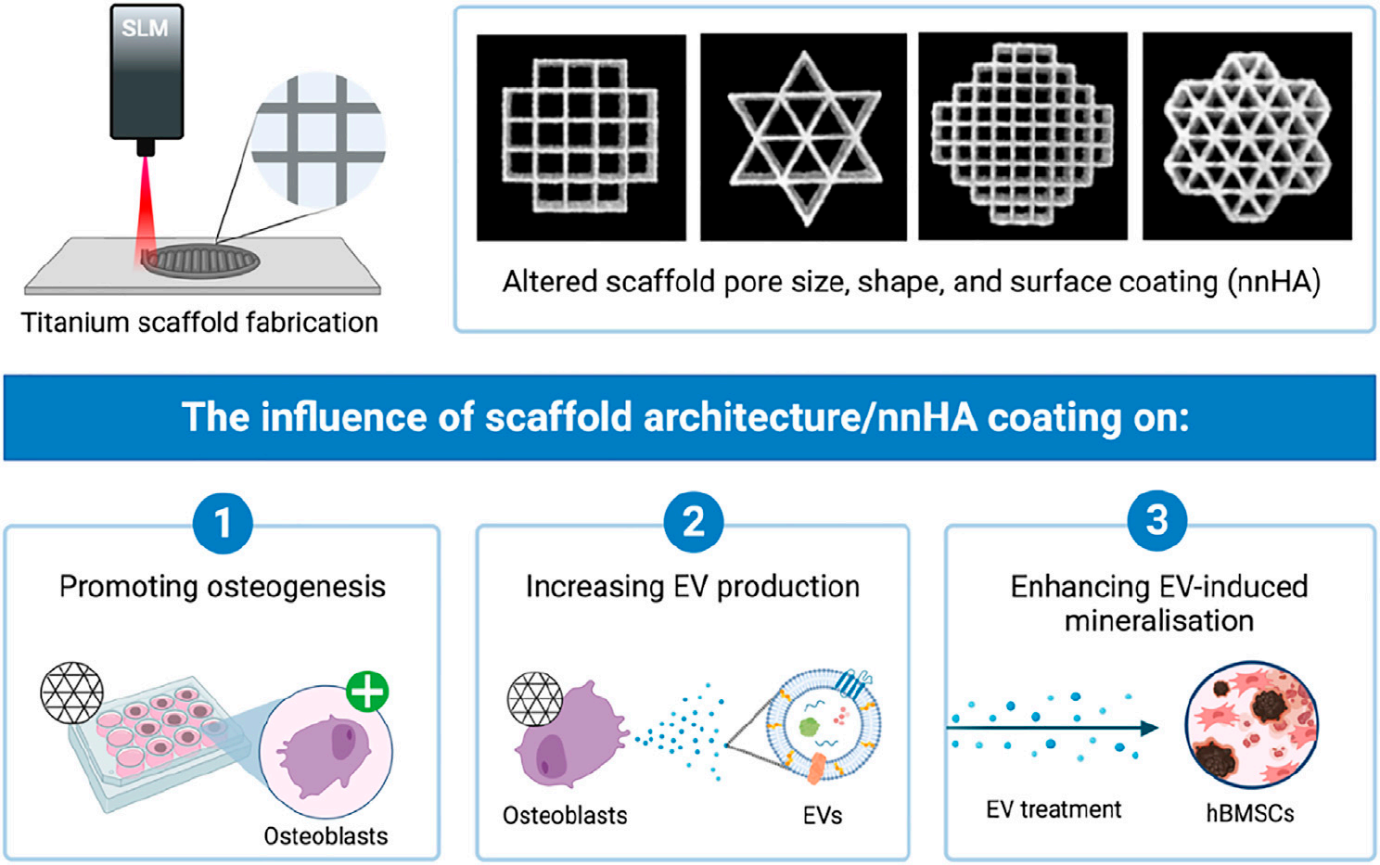


Cells are known to secrete a range of bioactive factors, which have been shown to regulate biological processes such as proliferation and differentiation through autocrine and paracrine signalling (Baraniak and McDevitt, 2010; Raposo and Stoorvogel, 2013; de Miguel-Gómez et al., 2020). One of these factors, Extracellular Vesicles (EVs), have gained increasing attention as novel acellular tools for regenerative medicine. EVs are cell-derived lipid nanoparticles that contain a diverse biological cargo including nucleic acids, proteins and bioactive molecules (Zaborowski et al., 2015; van Niel et al., 2018). The multitude of bioactive factors delivered by EVs has been shown to promote stem cell mineralisation when compared to the use of single growth factor treatment (Davies et al., 2017). Although the therapeutic efficacy of these cell-derived nanoparticles has been reported (Martins et al., 2016; Qin et al., 2016; Man et al., 2021a), to date EVs are commonly harvested from cells cultured on 2D tissue culture plastic. Conventional 2D culture limits the surface area for cell growth as well as providing an artificial cell-cell and cell-extracellular matrix interactions, which do not replicate *in situ* conditions mechanically or biologically (Abbott, 2003; Baker and Chen, 2012). By conferring a more physiologically relevant phenotype, it is hypothesised that this increased biomimicry will enhance the production of EVs exhibiting a more *in vivo*-like composition, improving their therapeutic potency for regenerative medicine (Thippabhotla et al., 2019; Man et al., 2020). Several studies have reported the influence of 3D culture on increasing the differentiation capacity of cells (Baraniak and McDevitt, 2012; Yamaguchi et al., 2014), however, limitations on current scaffold manufacturing hinders their implementation. Conventional scaffold fabrication techniques including solvent casting, freeze-drying and gas foaming provide limited control on scaffold architecture (Tabata, 2009), ultimately impacting the reproducibility of EVs from these systems. Moreover, there are issues associated with the procurement of EVs isolated from non-porous biomaterials, such as hydrogels (Patel et al., 2018), which may require extensive processing to extract these nanoparticles, ultimately damaging the integrity and functionality of the vesicles. Hence, there is a significant unmet need to refine the culture conditions to enhance EV therapeutic potency and yield for bone augmentation strategies.

In recent years, Additive Manufacturing (AM) techniques have facilitated the reproducible fabrication of scaffold systems that may more closely replicate the increased complexity of *in situ* environments (Gu et al., 2016; Li et al., 2016). Titanium-based materials have been broadly implemented in orthopaedic reconstruction as a result of their osteoinductivity and mechanical strength (Chen et al., 2017; Ilea et al., 2019). Selective Laser Melting (SLM) has allowed for the processing of titanium into 3D printed structures, increasing its clinical utility (Majumdar et al., 2018; Elsayed et al., 2019). Thus, the use of 3D printed titanium scaffolds could provide a more physiologically relevant platform for the manufacture of pro-regenerative EVs compared to conventional 2D tissue culture plastic. Although titanium has been extensively used for numerous orthopaedic applications, there have been numerous investigations aiming to improve its functionality by applying different surface finishes to promote osteoinduction (Damiati et al., 2018; Kligman et al., 2021). Consequently, several studies have explored the influence of applying surface coatings to 3D printed constructs to further promote their biomimetic nature (Kaur and Singh, 2019; Kazimierczak and Przekora, 2020). For example, Eichholz *et. al.* reported the use of a nano-needle hydroxyapatite (nnHA) coating to enhance the osteoinductive capacity of polycaprolactone (PCL) scaffolds fabricated by melt electrowriting (Eichholz et al., 2020a). Hence, modifying the scaffold’s surface composition could further improve the production of EVs. Together, this study aims to address pertinent issues hindering the clinical application of EVs, scalability of manufacture and therapeutic efficacy, thus facilitating the development of these instructive acellular tools to promote bone repair.

**FIGURE 1:**
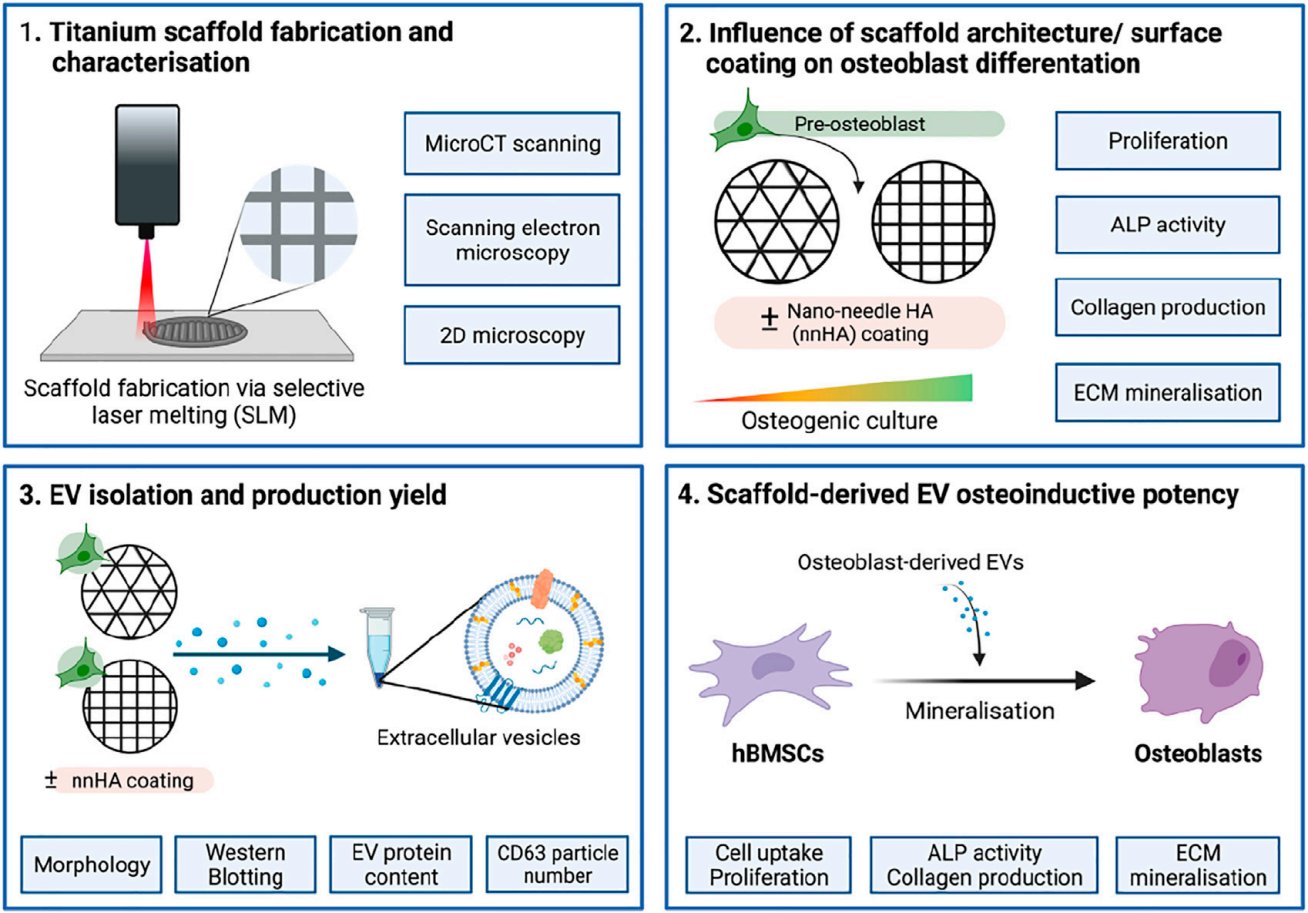
Schematic representation of study design. **1)** Titanium scaffolds were printed using SLM and their structure and topography were characterised. **2)** The effects of scaffold architecture and surface coating on osteoblast mineralisation were evaluated. **3)** EVs were isolated from scaffold-cultured osteoblasts and characterised. **4)** The osteoinductive potency of scaffold-derived EVs on stimulating hBMSC’s osteogenic differentiation was investigated. Created with BioRender.com.

In this present study, we investigated the influence of 3D printed scaffold architecture and surface composition on the therapeutic efficacy of osteoblast-derived EVs for bone repair. Titanium scaffolds were fabricated via SLM with differing pore sizes and shapes and determined their effects on osteoblast mineralisation (Figure 1). EVs were isolated from mineralising osteoblasts on different scaffold designs and administered to human bone marrow-derived mesenchymal stem cells (hBMSCs) to determine their efficacy in promoting osteogenic differentiation. Additionally, the effects of nnHA scaffold coating on osteoblast-derived EV yield and potency was evaluated.

## Materials and Methods

### Scaffold Fabrication and Processing

Lattice designs were created in CAD based on the designs from Van Bael *et. al.* (Van Bael et al., 2012) using Element (nTopology, Inc, United States), and slicing performed using QuantAM vs4 (Renishaw, PLC, United Kingdom). Four scaffolds were fabricated and classified according to shape [triangular (T) and square (S)] and pore size [500 µm (500) and 1000 µm (1000)]. The samples were manufactured on a RenAM 500 M selective laser melting system (Renishaw, PLC, United Kingdom) using gas atomised spherical Ti-6Al-4V grade 23 powder in the size range of 15 to 53 µm. The slice thickness was 30 μm, the laser power was 100 W for the contours and 200 W for the hatched regions, and the scanning speed was equivalent to 1125 mm/s for the contours and 1100 mm/s for the hatched regions. The samples were removed from the build substrate using wire electro-discharge machining, Cut20 (Beijing Agie Charmilles. Ltd, China). The scaffolds were ultrasonically cleaned in acetone, 95% ethanol and distilled water for 15 min each, then incubated in 5 M NaOH for 24 h at 60°C. Following which, the samples were sterilised by autoclave prior to cell culture.

### Nano-Needle HA Coating

A nnHA coating was applied to the scaffolds as previously described (Eichholz et al., 2020a). Briefly, the autoclaved samples were immersed in 70% ethanol at room temperature under vacuum to remove air bubbles. Scaffolds were then incubated with 2 M NaOH for 45 min at 37°C, rinsed in MilliQ water five times and then incubated with calcium solution (0.05 M Calcium Chloride Dihydrate (Sigma-Aldrich, United Kingdom) in MilliQ water). An equal volume of phosphorus solution, (0.03 M Sodium Phosphate Tribasic Dodecahydrate (Sigma-Aldrich, United Kingdom) in MilliQ water), was slowly added to the calcium solution. Air bubbles were removed under a vacuum, and samples were incubated for 30 min at 37 C. The coating procedure was repeated twice, with fresh solutions used each time. Samples were then incubated with 0.5 M NaOH for 30 min at 37°C and rinsed with MilliQ water five times and allowed to air-dry overnight. Samples were UV sterilised for 20 min on each side prior to cell culture. Scaffolds coated with nnHA were designated T500-H and T1000-H.

### Scanning Electron Microscopy (SEM)

The morphology of the scaffold pores was examined on randomly selected samples using a ZEISS EVO MA10 scanning electron microscope (ZEISS, Germany) operating at 15 kV.

### Microcomputed Tomography Analysis

Microcomputed tomography (micro-CT) scans were taken with a SkyScan 1172 (Bruker), using the following settings: aluminium-copper filter, current 100 mA, voltage 80 kV, exposure time 800 ms, pixel size 11.9 μm, camera resolution 1000 × 666 pixels, rotation step 0.6°, frame averaging 4. Scans were reconstructed using NRecon (version 1.6.10.2, Bruker), and 3D models were produced in CTVox (version 3.0.0, Bruker). The same scanning, reconstruction, and post-reconstruction parameters were used for all scans. Micro-CT image analysis was used to calculate the strut and pore size distributions, porosity, surface area, volume and interconnectivity. The permeability coefficients in the horizontal and vertical directions of the different designs were calculated using the protocol reported by Van Bael *et al.* (Van Bael et al., 2012).

### Cell Culture and Reagents

MC3T3 murine pre-osteoblasts were purchased from American Type Culture Collection (ATCC, United Kingdom) and hBMSCs were acquired from Lonza (Lonza, United Kingdom). Basal culture media consisted of minimal essential medium (α-MEM; Sigma-Aldrich, United Kingdom) supplemented with 10% Foetal Bovine Serum (FBS), 1% penicillin/streptomycin (Sigma-Aldrich, United Kingdom) and L-glutamine (Sigma-Aldrich, United Kingdom). hBMSCs were used at passage 4. Mineralisation medium comprised of basal culture media supplemented with 10 mM β-glycerophosphate (Sigma-Aldrich, United Kingdom) and 50 μg/mL L-ascorbic acid (Sigma-Aldrich, United Kingdom). Culture medium utilised for EV isolation and dosing was depleted of FBS-derived EVs by ultracentrifugation at 120,000 g for 16 h prior to use.

The sterilised scaffolds were washed three times with sterile Phosphate Buffered Saline (PBS) and incubated overnight with basal medium at 37°C. Cells (2 × 10^5^ cells) were statically seeded onto each scaffold for 1 h at 37°C. Following this, the samples were loaded onto an SB tube rotator (SB3, STUART, United Kingdom) and dynamically cultured for 16 h at 8 rpm. The scaffolds were then removed and washed with basal media twice, before incubation in osteogenic media. Cells cultured on 2D tissue culture plastic were used as control.

Cell viability on titanium constructs was assessed via live/dead staining. Samples were incubated in basal medium containing CellTrackerTM Green CMFDA (2 μM, Thermo Scientific, United Kingdom) and Ethidium homodimer-1 (4 μM, Sigma-Aldrich, United Kingdom) for 30 min. The medium was replaced with fresh medium for 30 min before visualised under an EVOS fluorescent inverted microscope (M5000, Thermo Scientific, United Kingdom).

Histone Deacetylase (HDAC) activity was assessed using the fluorometric HDAC assay kit (Abcam, United Kingdom) according to the manufacturer’s protocol. Relative HDAC activity was determined in a SPARK spectrophotometer with an excitation and emission of 350 and 440 nm respectively. HDAC activity normalised with protein content. Detection of H3K9 acetylation was performed using the EpiQuik Global Histone H3 Acetylation Assay Kit (Epigentek, United States) according to the manufacturer’s protocol. The absorbance was read in a SPARK spectrophotometer at 450 nm. Histone acetylation was normalised with protein content.

Relative gene expression was evaluated as previously described (Man et al., 2021a). Briefly, total RNA was extracted using the RNase mini kit (Qiagen, United Kingdom) from scaffold-cultured osteoblasts according to the manufacturer’s protocol. Primers (Supplementary Table S1) (Primerdesign, United Kingdom) were used to quantify levels of alkaline phosphatase (*ALP*), collagen type I (*COL1A*), and osteocalcin (*OCN*). Glyceraldehyde 3-phosphate dehydrogenase (*GAPDH*) was used as the internal reference. RNA was amplified in a 20 μL reaction with a 96-well PCR plate (Starlab, United Kingdom). Amplification occurred using the AriaMx Real-Time PCR System (Agilent Technologies, United Kingdom). The cycle threshold (Ct) value was acquired and the comparative Ct method (2^-∆∆Ct^) was utilised to quantify the gene expression levels relative to the housekeeping gene.

### EV Isolation and Characterisation

#### EV Isolation

Medium was isolated from osteoblasts cultured on titanium scaffolds every 2 days for 14 days. EVs were isolated from the collected conditioned medium by differential centrifugation as previously described (Man et al., 2021a): 2000 g for 20 min, 10,000 g for 30 min and 120,000 g for 70 min. The EV pellet was washed with sterile PBS and centrifuged at 120,000 g for 70 min and the resultant pellet was re-suspended in 500 μL PBS. All ultracentrifugation steps were performed utilising the Sorvall WX Ultra Series Ultracentrifuge (Thermo Scientific, United Kingdom) and a Fiberlite, F50L-8×39 fixed angle rotor (Piramoon Technologies Inc., United States).

#### Particle Size and Concentration Analysis

Total EV protein concentration was determined using the Pierce BCA protein assay kit (Thermo Scientific, United Kingdom). The quantity of CD63 positive particles was quantified by using ExoELISA-ULTRA CD63 Kit (System Biosciences, United States). EV protein and CD63 particle concentration were normalised with cell number. Dynamic Light Scattering (DLS) (Zetasizer Nano ZS, Malvern Instruments, United Kingdom) was used to analyse size distribution and zeta potential.

#### Immunoblotting

Immunoblotting was used to confirm the presence of EVs as previously described (Man et al., 2021a). Briefly, EV proteins from osteoblasts cultured on 2D or T1000 constructs were electrophoretically separated using precast gels (4–15% Mini-PROTEAN TBX, Biorad, United Kingdom). Gels were then blotted on polyvinylindene difluoride membranes (Fisher Scientific, United Kingdom) and blocked with EveryBlot blocking buffer (BioRad, United Kingdom). Primary antibodies to Alix (1:1000 dilution, Santa Cruz, United States), Annexin 2 (1:2000 dilution, Abcam, United Kingdom), CD9 (1:1000 dilution, Abcam, United Kingdom) and calnexin (1:1000 dilution, Abcam, United Kingdom) were incubated with the blot overnight at 4 C. The membranes were then incubated with the appropriate secondary antibody, anti-mouse for Alix (1:3000 dilution, Cell Signaling, United Kingdom), and anti-rabbit for Annexin 2, CD9 and calnexin (1:3000 dilution, Cell Signaling, United Kingdom), for 1 h at room temperature. Chemiluminescence detection of bands were imaged with the ChemiDoc XRS + system (BioRad, United Kingdom) using Clarity™ Western ECL substrate (BioRad, United Kingdom) and Image Lab software (Life Science Research, BioRad, United Kingdom) following the manufacturer’s protocol.

#### Transmission Electron Microscopy

Isolated EVs were visualised using a JEOL JEM1400 transmission electron microscope (TEM, JEOL, United Kingdom) coupled with an AMT XR80 digital acquisition system. Prior to imaging, EVs were physisorbed to a 200-mesh carbon-coated copper formvar grid (Agar Scientific, United Kingdom) and negatively stained with 1% uranyl acetate.

### hBMSCs Treatment With EVs

#### EV Cell Uptake

EVs were labelled using Cell Mask™ Deep Red Plasma Membrane Stain, 1:1000 in PBS, (Thermo Scientific, United Kingdom) and incubated for 10 min. Labelled EVs were washed twice with PBS via ultracentrifugation at 120,000 g for 70 min hBMSCs were seeded (4 × 10^3^ cells/cm^2^) in a chamber slide (Corning, United Kingdom) for 24 h then media was replaced with fresh basal media supplemented with labelled EVs. After 24 h, cells were fixed with 10% (v/v) neutral buffered formalin (NBF, Cellpath, United Kingdom), stained with Alexa Fluor 488 phalloidin, 1:20, (Cell Signalling Technology, United Kingdom) and mounted with Prolong™ Gold Antifade Mountant with DAPI (Thermo Scientific, United Kingdom) to label the actin cytoskeleton and nuclei respectively. Slides were imaged with an EVOS fluorescent inverted microscope (M5000, Thermo Scientific, United Kingdom).

#### hBMSCs Osteogenic Culture

Cells were seeded to a density of 21 × 10^3^ cells/cm^2^ in 24-well plates (Nunc, United Kingdom) with basal medium for 24 h. The medium was replaced with fresh mineralisation medium supplemented with EVs (5 μg/ml of EV protein) for 21 days. EV-supplemented medium changes were performed every 48 h and cells cultured in mineralising medium alone was used as the control.

#### Intracellular Calcium Activity

Intracellular calcium content was quantified using the Calcium Colorimentic Assay Kit (Sigma, United Kingdom) following the manufacturer’s protocol. Briefly, 50 μL of cell lysate was incubated with 90 μL of Chromogenic reagent and 60 μL of Calcium assay buffer. Samples were incubated at room temperature for 10 min and absorbances were read at 575 nm on a SPARK spectrophotometer (TECAN, CH). For the nnHA samples, cell-free coated scaffolds were incubated in osteogenic medium for 2 weeks and used as the blank readings.

### Alkaline Phosphatase Activity

ALP activity was determined using the 4-nitrophenyl colourimetric phosphate liquid assay (pNPP, Sigma-Aldrich, United Kingdom) as previously reported (Man et al., 2021b). Briefly, 10 μL of cell lysate (in 0.1% Triton™ X-100) was added to 90 μL of pNPP and incubated for 60 min at 37 C. The absorbance at 405 nm was read on a SPARK spectrophotometer (TECAN, CH). ALP activity was normalised with DNA content.

### DNA Quantification

DNA content was determined using the Quant-iT PicoGreen DNA assay (Invitrogen, Life Technologies, United Kingdom). Briefly, 10 μL of cell lysate (in 0.1% Triton™ X-100) was added to 90 μL of TE (10 mM Tris-HCl, 1 mM EDTA) buffer. 100 μL of PicoGreen reagent was added to all samples for 5 min. The fluorescence was then measured in a SPARK spectrophotometer (TECAN, CH) at 480/520 nm wavelength.

### Collagen Production

Collagen deposition was evaluated with picrosirius red staining. Briefly, cells were washed twice in PBS and fixed in 10% NBF for 30 min, prior to staining with Picro-Sirius Red Solution (ScyTek Laboratories, Inc., United States) for 1 h. The unbound dye was removed by washing in 0.5 M acetic acid followed by distilled water wash and left to air dry prior to imaging using light microscopy (EVOS XL Core, Invitrogen, United Kingdom). To quantify collagen staining, 0.5 M sodium hydroxide was used to elute the bound dye and absorbance read at 590 nm using a SPARK spectrophotometer (TECAN, CH).

### Calcium Deposition

Alizarin red staining was conducted to evaluate calcium deposition. Briefly, cells were washed twice in PBS and fixed in 10% NBF for 30 min. Following fixation, cells were washed in distilled water and then incubated with alizarin red solution (Sigma-Aldrich, United Kingdom) for 10 min. The unbound dye was removed by washing in distilled water. Staining was visualised using light microscopy (EVOS XL Core, Invitrogen, United Kingdom). For alizarin red quantification, stained samples were eluted with 10% cetylpyridinium chloride (Sigma-Aldrich, United Kingdom) for 1 h and then absorbance read at 550 nm using the SPARK spectrophotometer (TECAN, CH).

### Statistical Analysis

For all data presented, experiments were performed in triplicate. All statistical analysis was undertaken using ANOVA multiple comparisons test followed by Tukey’s post hoc using IBM SPSS software (IBM Analytics, version 21). *p* values equal to or lower than 0.05 were considered as significant. *P ≤ 0.05, **P ≤ 0.01 ***P ≤ 0.001.

## Results

### Influence of Titanium Scaffold Morphological Architecture on Osteoblast Mineralisation

**FIGURE 2:**
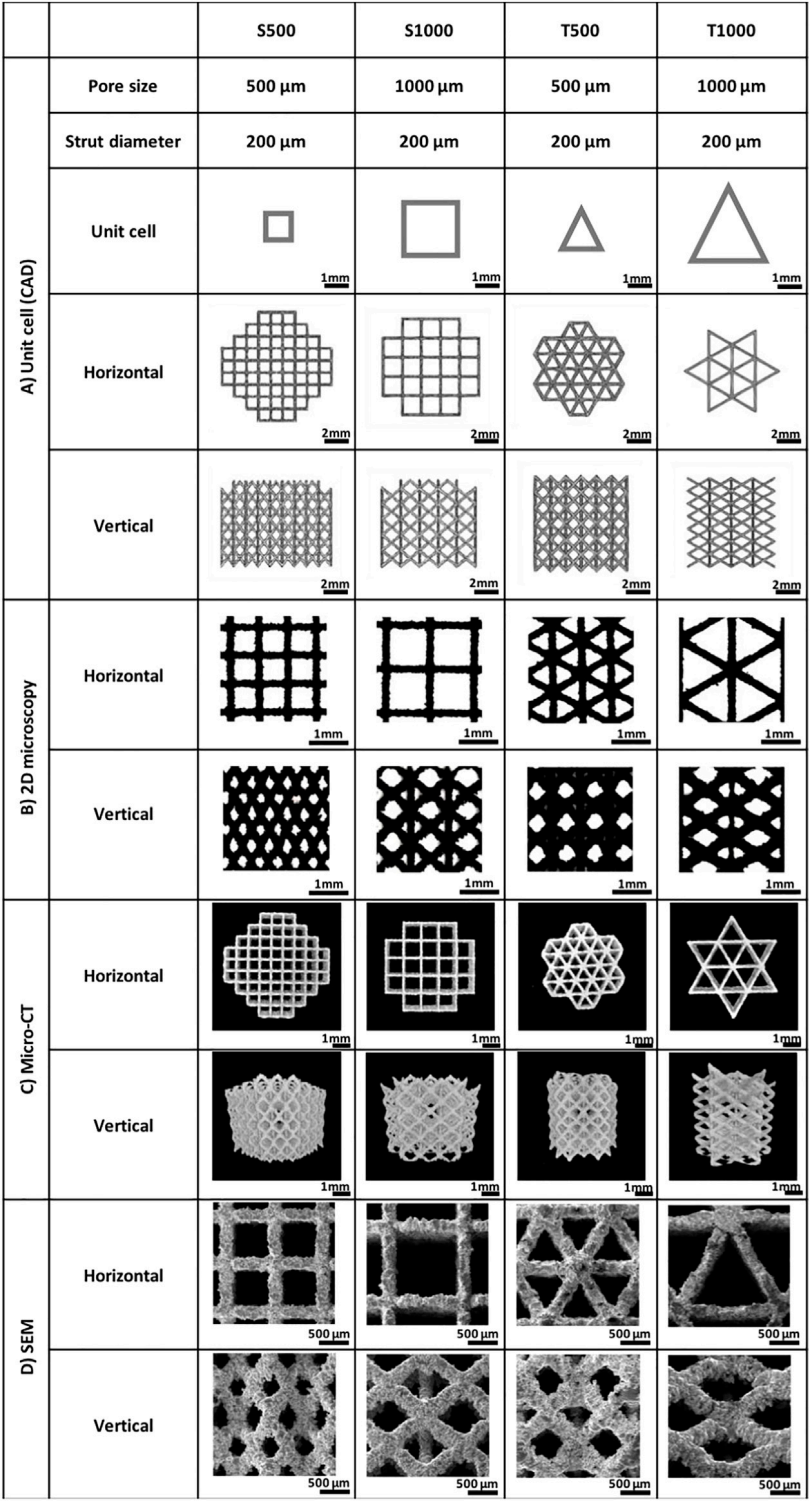
Morphological analysis of the titanium scaffolds manufactured for the current study including **(A)** CAD unit cells, **(B)** 2D brightfield microscopy, **(C)** micro-CT scans and **(D)** SEM images. Scaffold designation **=** triangular (T) and square (S) and pore size (500 µm (500) and 1000 µm (1000)).

Scaffolds exhibiting either a square or triangle pore shape, with pore sizes of 500 or 1000 µm were fabricated using SLM. To visualise the scaffolds geometry, the CAD models (Figure 2A) and images from the produced scaffolds were obtained via 2D microscopy (Figure 2B), micro-CT scanning (Figure 2C) and SEM analysis (Figure 2D). The fabricated scaffolds pore and strut sizes were measured by 2D light microscopy and compared to the theoretical CAD values (Table 1). There was a high degree of similarity in the pore (>98%) and strut (>96%) measurements between the designed and fabricated values. Moreover, micro-CT analysis demonstrated that changing scaffold pore size and morphology affected key scaffold properties such as surface area, structure volume and porosity. Additionally, permeability analysis confirmed the influence of increased pore size and triangle pore conformation on enhancing fluid flow through these scaffolds.

**TABLE 1:** Characterisation of titanium scaffolds fabricated by SLM.

	S500	S1000	T500	T1000
Pore size (µm)	512 ± 17.7	999 ± 1.6	511 ± 16.1	989 ± 8.1
Strut size (µm)	203.4 ± 1.3	203 ± 2.6	207 ± 3.4	206 ± 3.5
Surface area (mm^2^)	474 ± 8	306 ± 4	262 ± 6	163 ± 2
Structure volume (mm^3^)	64 ± 2	33 ± 1	53 ± 3	28 ± 2
Porosity (%)	77.34	87.44	67.94	80.42
Interconnectivity (%)	100	100	100	100
Permeability coefficient (Horizontal direction)	4.04	21.06	7.07	31.42
Permeability coefficient (Vertical direction)	1.42	3.84	1.95	7.79

**FIGURE 3:**
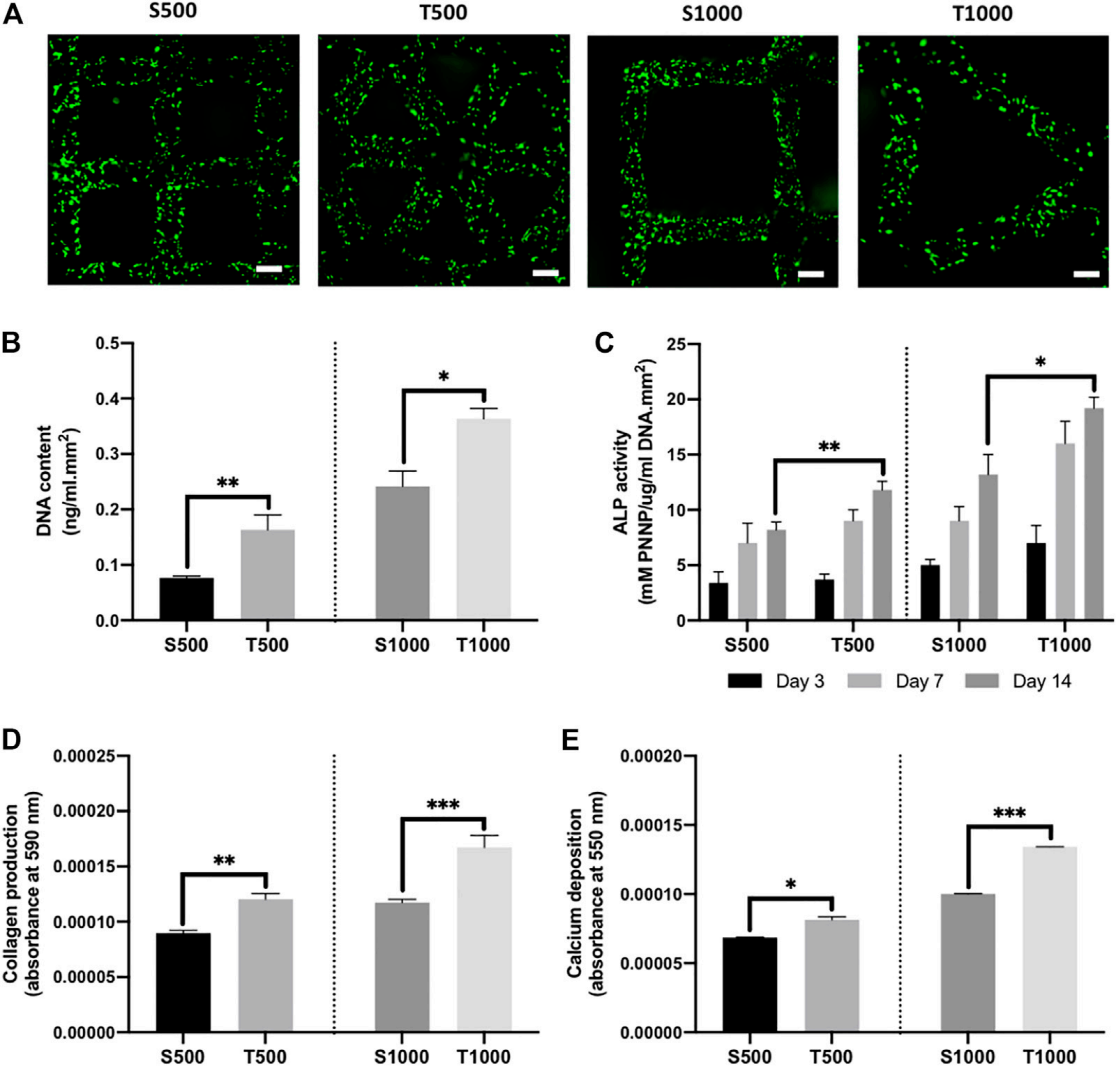
Influence of titanium scaffold architecture on osteoblast behaviour and mineralisation. **(A)** Live/dead staining of osteoblasts cultured on titanium scaffolds following dynamic seeding, scale bar = 200 µm. The effects of scaffold design on osteoblast **(B)** DNA content, **(C)** ALP activity, **(D)** collagen production and **(E)** calcium deposition following 14 days osteogenic culture. Data are expressed as mean ± SD (n = 3). *P ≤ 0.05, **P ≤ 0.01 and ***P ≤ 0.001.

Following dynamic seeding of osteoblasts on the titanium scaffolds, viable cells (green) were observed evenly distributed on the scaffold struts 24 h post-seeding (Figure 3A). There was a significant increase in the DNA content from osteoblasts cultured on triangle pore scaffolds when compared to the square pore lattices after 14 days of culture, where the T500 and T1000 constructs exhibited a 2.13- (P ≤ 0.01) and 1.5-fold (P ≤ 0.05) higher DNA content when compared to the S500 and S1000 scaffolds, respectively (Figure 3B). To determine the influence of scaffold design on osteoblast epigenetic functionality, HDAC activity and histone acetylation was quantified following 7 days of osteoinductive culture (Supplementary Figure S1). There was a significant reduction in HDAC activity in the triangle pore scaffolds when compared to the square pore constructs of the same size (P ≤ 0.05–0.01). Moreover, we determined that the decrease in HDAC activity resulted in a significant increase in histone acetylation levels within the cells cultured on the triangle pore scaffolds (P ≤ 0.001). The effects of scaffold architecture on osteoblast osteogenic differentiation was initially evaluated by quantifying osteogenic gene expression (Supplementary Figure S2). With increasing pore size and triangle pore shape, the expression of osteoblast-related markers ALP, COL1A and OCN were significantly upregulated when compared to cells cultured on the smaller pore scaffolds with a square pore conformation (P ≤ 0.01–0.001). There was a time-dependent increase in ALP activity in all scaffold groups during culture (Figure 3C). At day 14, a significantly elevated ALP activity was observed in the T500 (1.43-fold, P ≤ 0.01) and T1000 (1.45-fold, P ≤ 0.05) groups compared to the square pore scaffolds of the same pore size. A similar trend was observed in the collagen and calcium deposition analysis. Cells cultured on the T500 and T1000 scaffolds exhibited a significant 1.35- (P ≤ 0.01) and 1.43-fold (P ≤ 0.001) increased in collagen production, and a 1.19- (P ≤ 0.05) and 1.3-fold (P ≤ 0.001) enhancement in calcium deposition when compared to the S500 and S1000 scaffolds respectively, following 14 days of osteoinductive culture (Figures 3D,E).

### The Effect of Scaffold-Derived EVs on hBMSCs Osteogenic Differentiation and Mineralisation

**FIGURE 4:**
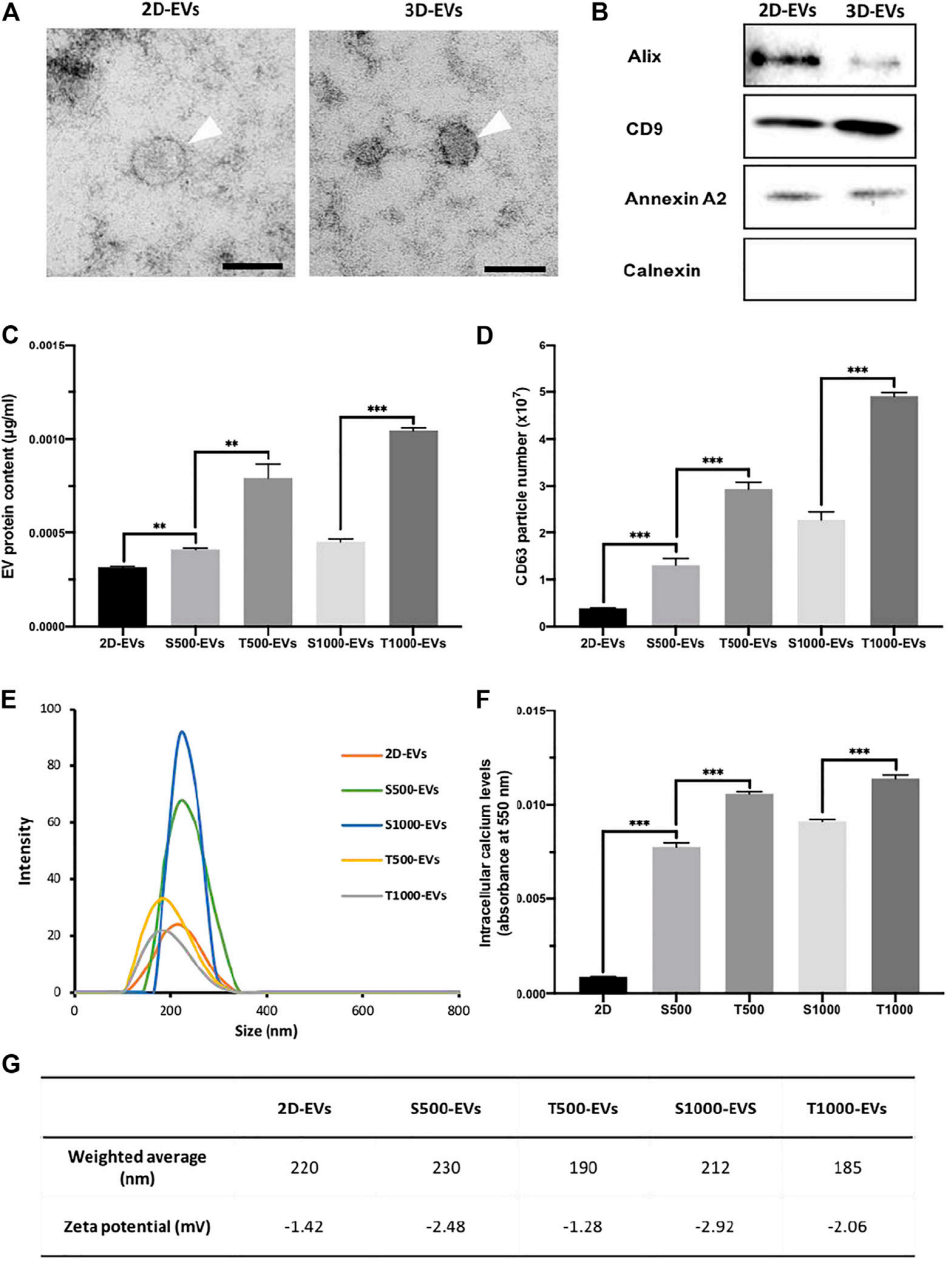
Characterisation of osteoblast-derived EVs: **(A)** representative TEM image of EVs derived from 2D and 3D culture, scale bar = 50 nm, **(B)** western blot analysis, **(C)** EV protein content, **(D)** CD63^+^ particles quantification, **(E)** size distribution, **(F)** intracellular calcium content and **(G)** weighted average diameter and zeta potential of isolated EVs. Data are expressed as mean ± SD (n = 3). **P ≤ 0.01 and ***P ≤ 0.001.

Initially, EVs were isolated from scaffold-cultured osteoblast conditioned media via ultracentrifugation, and the nanoparticles were then characterised. TEM imaging showed that EVs derived from scaffold-cultured cells exhibited a typical spherical morphology, similar to the EVs procured from 2D-cultured osteoblasts (Figure 4A). EVs derived from 2D and scaffold-cultured osteoblasts exhibited positive EV marker expression (Figure 4B). EV protein content was significantly enhanced (>1.32-fold, P ≤ 0.01) from osteoblasts cultured on scaffolds when compared to 2D (Figure 4C). The T500 and T1000 groups exhibited a 1.93- and 2.22-fold increase when compared to the S500 (P ≤ 0.01) and S1000 (P ≤ 0.001) scaffolds respectively. To ascertain whether protein content quantified was EV specific, the concentration of CD63 positive particles were evaluated *via* the CD63 ELISA. There was a >3.4-fold increase in CD63 positive particles derived from the scaffold-cultured osteoblast compared to 2D culture (P ≤ 0.001) (Figure 4D). The T500 and T1000 groups exhibited a 2.24- and 2.15-fold increase in CD63 positive particles when compared to the S500 (P ≤ 0.001) and S1000 (P ≤ 0.001) scaffolds respectively. Isolated EVs exhibited an average diameter around 200 nm (Figures 4E,G) and a negative surface change (Figure 4G). Intracellular calcium content has been causatively linked to EV biogenesis, as such we quantified the calcium levels from scaffold-cultured osteoblasts. The findings showed a significantly elevated levels of intracellular calcium from scaffold-cultured cells when compared to the cells from 2D (>9.1-fold) (P ≤ 0.001), with the T500 (1.36-fold) and T1000 (1.27-fold) groups exhibited increased intracellular calcium when compared to the S500 and S1000 respectively (P ≤ 0.001) (Figure 4F).

**FIGURE 5:**
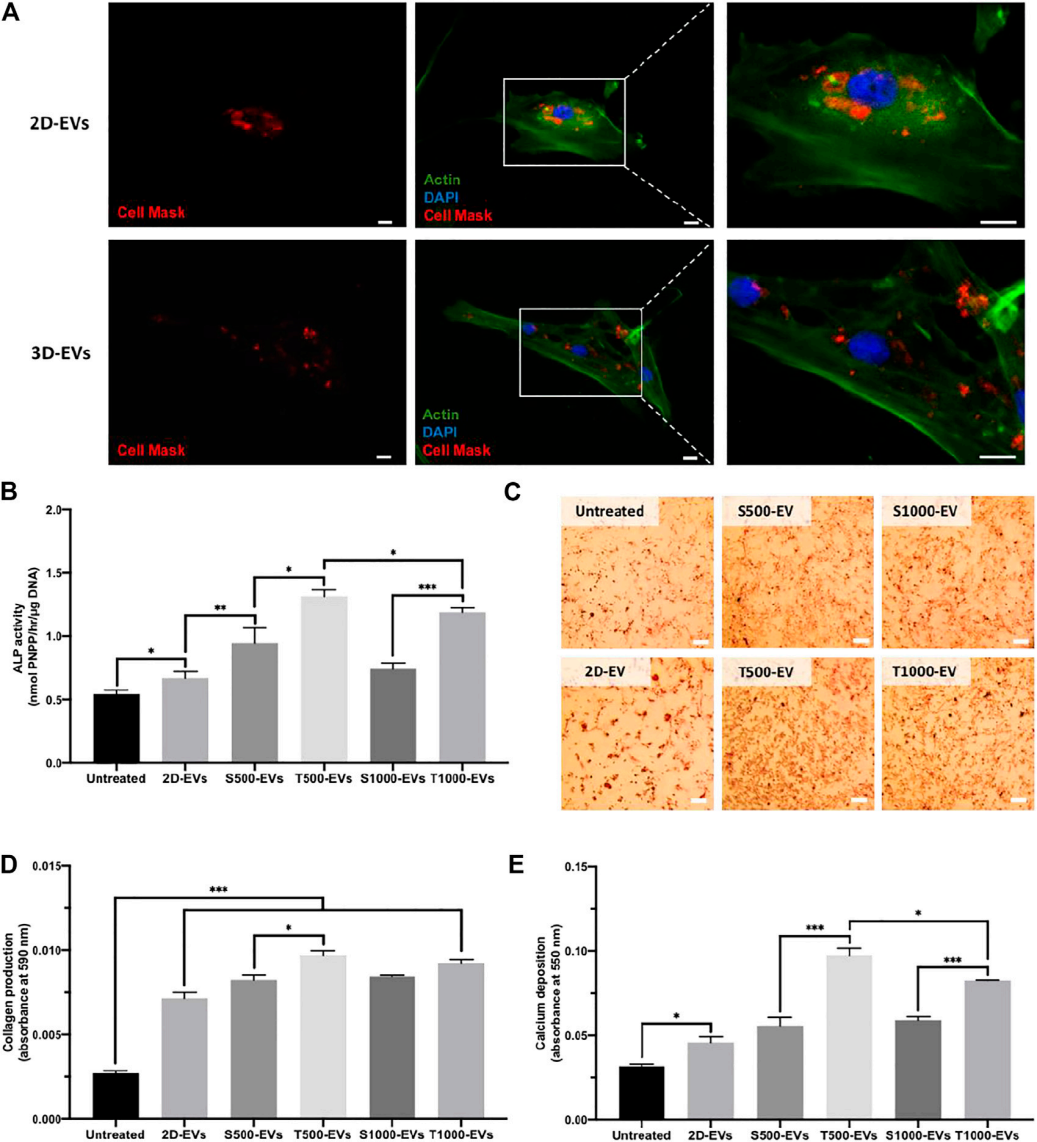
Effect of scaffold-derived osteoblast EVs on hBMSC’s osteogenic differentiation and mineralisation. **(A)** Osteoblast-derived EV cell uptake. Scale bar = 20 µm. **(B)** ALP activity, **(C,D)** collagen production **(E)** calcium deposition following 21 days osteogenic culture. Scale bar = 200 µm. Data are expressed as mean ± SD (n = 3). *P ≤ 0.05, **P ≤ 0.01 and ***P ≤ 0.001.

Cell Mask labelled EVs derived from 2D and scaffold cultures were successfully internalised by hBMSCs following 24 h incubation, with labelled EVs situated within the cell’s cytoplasm (Figure 5A). The influence of scaffold architecture on osteoblast-derived EV osteogenic potency was initially evaluated by quantifying hBMSCs ALP activity (Figure 5B). 2D-EVs and 3D-EVs significantly increased hBMSCs ALP activity when compared to the untreated cells after 14 days osteogenic culture. The T500-EV and T1000-EV treatment significantly enhanced ALP activity when compared to the S500-EV (1.39-fold, P ≤ 0.05) and S1000-EV (1.59-fold, P ≤ 0.001) treated hBMSCs, respectively, with the T500-EVs eliciting a slight significant increase in ALP activity compared to the T1000-EV group (P ≤ 0.05). The influence of scaffold-derived EVs on hBMSCs extracellular matrix production and mineralisation was assessed by quantifying collagen content and calcium deposition, respectively. Similar to the findings in ALP activity, collagen production was significantly enhanced in the EV treated cells compared to the untreated control (>2.63-fold, P ≤ 0.001) (Figures 5C,D). The T500 and T1000 groups exhibited an increase in collagen production compared to the S500 (1.21-fold, P ≤ 0.05) and S1000 (1.15-fold, P > 0.05) groups, respectively. Alizarin red staining showed that the 2D-EVs (1.44-fold, P ≤ 0.05) and scaffold-derived EVs (>1.76-fold, P ≤ 0.05–0.001) treatment significantly enhanced hBMSCs calcium deposition compared to the untreated cells after 21 days of osteogenic culture (Figure 5E). Between the scaffold-EV treated groups, the T500-EV and T1000-EV treated hBMSCs exhibited significantly increased calcium deposition when compared to the S500-EV (1.75-fold, P ≤ 0.001) and S1000-EV (1.4-fold, P ≤ 0.001) treated cells. Additionally, the T500-EVs significantly increased hBMSCs calcium deposition when compared to the T1000-EV treated group (P ≤ 0.05).

### Influence of Bone-Mimetic nnHA Surface Treatment on Osteoblast Mineralisation and Osteoblast-Derived EV Osteoinductive Efficacy

**FIGURE 6:**
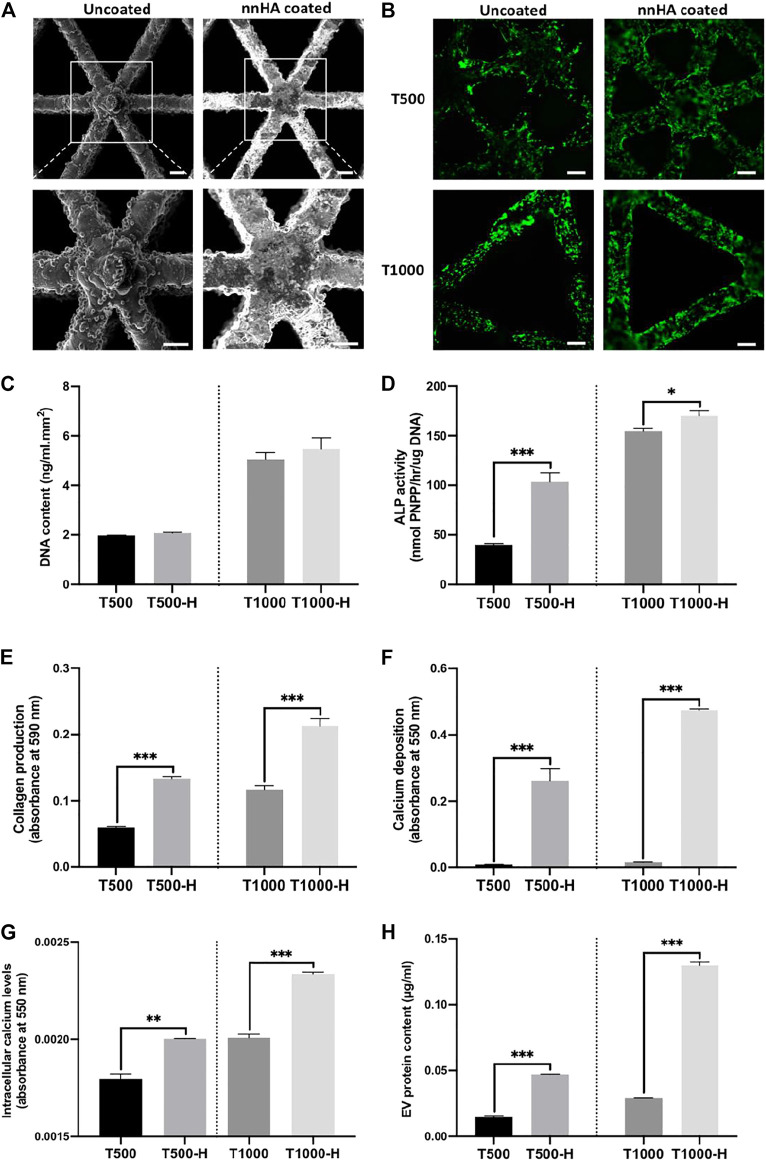
The impact of nnHA coating on scaffold-cultured osteoblast mineralisation and EV production. **(A)** Representative SEM image of uncoated and nnHA coated titanium scaffold, scale bar = 200 μm, **(B)** live-dead staining of osteoblast following 24 h of culture, Scale bar = 200 μm, **(C)** proliferation, **(D)** ALP activity, **(E)** collagen content, **(F)** calcium deposition, **(G)** intracellular calcium levels and **(H)** EV procurement yield. Data are expressed as mean ± SD (n = 3). *P ≤ 0.05, **P ≤ 0.01 and ***P ≤ 0.001.

Triangle pore scaffolds were coated with or without a nnHA coating to evaluate its influence on osteoblast mineralisation and osteoblast-derived EV osteoinductive potency. Figure 6A shows a representative SEM image of uncoated and nnHA coated titanium scaffolds, with mineral deposition observed on the latter. Alizarin red staining confirmed the presence of calcium on the nnHA coated surface (Supplementary Figure S3). Following dynamic seeding, viable osteoblasts (green) can be observed evenly distributed over the scaffolds surface (Figure 6B). There was a significant increase in the DNA content on the larger pore sized scaffolds (T1000, T1000-H), when compared to the scaffolds exhibiting the smaller pore size (T500, T500-H) (>2.44-fold, P ≤ 0.001) (Figure 6C). Between the untreated and nnHA coated scaffolds of the same pore size, there was a slight non-significant increase in the DNA content on the nnHA coated lattices compared to the uncoated control (P > 0.05). The effects of nnHA scaffold coating on osteoblast osteogenic differentiation were evaluated through quantifying ALP activity, collagen production and calcium deposition. The T500-H and T1000-H scaffolds significantly increased osteoblast osteogenic differentiation and mineralisation after 14 days in osteogenic culture when compared to the T500 and T1000 scaffolds respectively (P ≤ 0.05–0.001) (Figures 6D–F). Cells cultured on the T500-H and T1000-H scaffolds exhibited a significantly increased intracellular calcium content compared to the T500 (1.18-fold, P ≤ 0.01) and T1000 constructs (1.15-fold, P ≤ 0.001) (Figure 6G). Additionally, the nnHA scaffold cultured osteoblast secreted a significantly enhanced quantity of EVs during culture when compared to the untreated controls (3.5, 4.45-fold, P ≤ 0.001) (Figure 6H).

**FIGURE 7:**
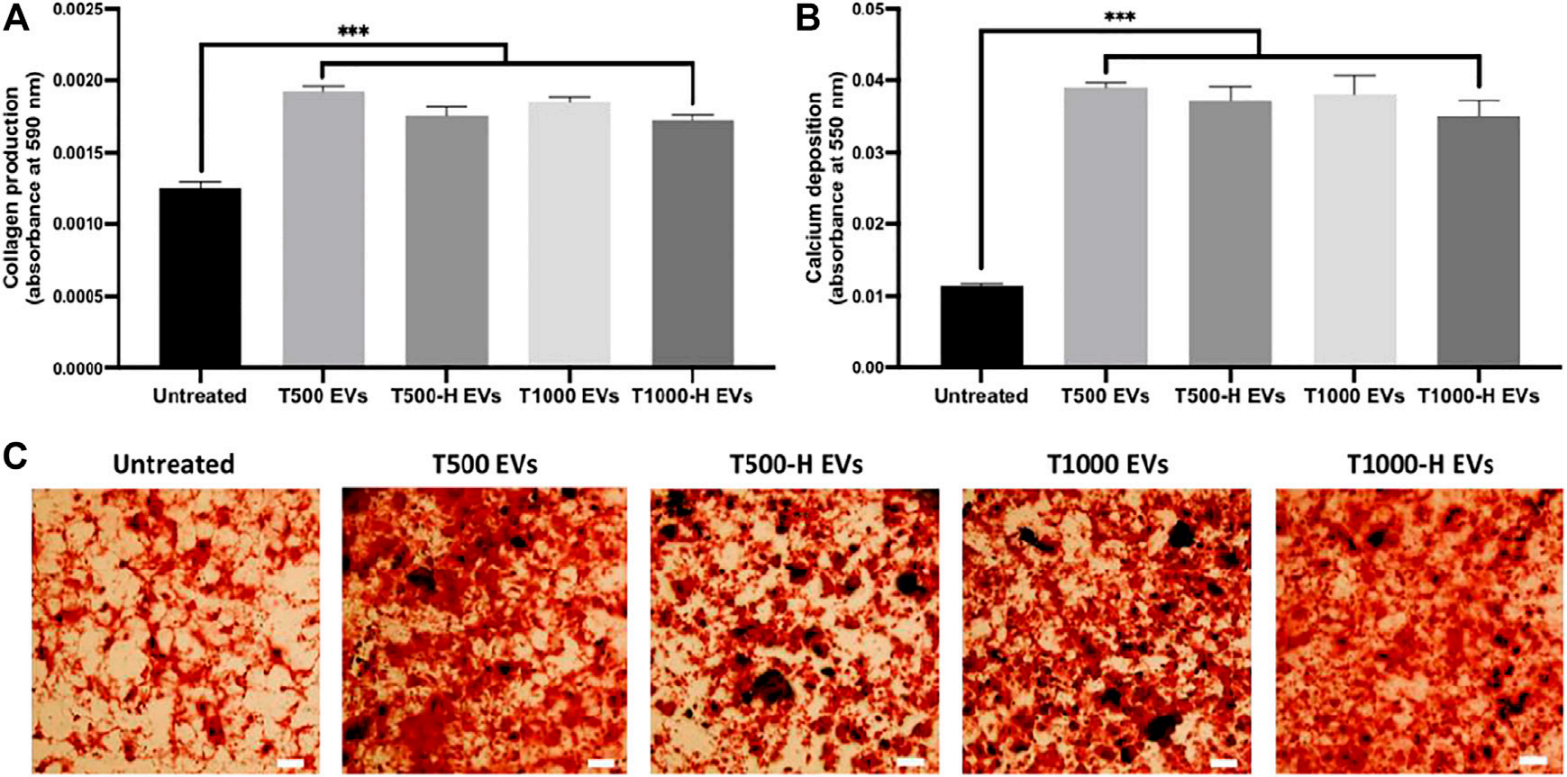
The influence of nnHA scaffold coating on osteoblast-derived EVs osteoinductive efficacy. **(A)** Collagen production and **(B)**, **(C)** calcium deposition following 21 days of osteogenic culture. Black staining indicates mineral nodules. Scale bar = 200 µm. Data are expressed as mean ± SD (n = 3). ***P ≤ 0.001.

The influence of nnHA coating on osteoblast-derived EV osteogenic potency was evaluated by assessing hBMSCs osteogenesis. EVs derived from uncoated and nnHA coated scaffolds significantly increased hBMSCs extracellular matrix collagen production (>1.38-fold) (Figure 7A) and calcium deposition (>3.18-fold) (Figures 7B,C) when compared to the untreated cells (P ≤ 0.001), with no significant differences observed between the EV groups (P > 0.05).

## Discussion

There is growing precedence to utilise 3D culture platforms to more closely replicate *in vivo* environments, ultimately improving the biomimicry of EVs isolated from *in vitro* systems (Man et al., 2020). In this regard, harnessing AM methods such as 3D printing would allow for the large-scale, reproducible fabrication of 3D constructs with precise control on scaffold parameters, overcoming limitations of conventional scaffold systems for EV procurement (Tabata, 2009; Patel et al., 2018). Moreover, several studies have reported the osteoinductive capacity of titanium substrates (Xu et al., 2013; Salou et al., 2015), thus possibly providing a pro-osteogenic platform for EV manufacture. Therefore, this study aimed to address pertinent issues hindering the clinical translation of EVs, scalability of manufacture and therapeutic efficacy, hence facilitating the development of EVs as novel acellular therapies to promote bone repair.

It has become increasingly apparent that the mechanobiological interactions between cells and their substrates play a vital role in augmenting the cell’s functionality (Guilak et al., 2014). Numerous studies have shown the importance of pore geometry in modifying the differentiation capacity of cells (Van Bael et al., 2012; Xu et al., 2014; Rubert et al., 2021). In this study, constructs exhibiting a triangle pore conformation significantly improving osteoblast differentiation and mineralisation when compared to square pored scaffolds of both sizes. It has been similarly reported in the literature that a more acute 3D printed fibre angle promotes cellular contact, increasing the cell’s osteogenic capacity (Guvendiren et al., 2017; Ji and Guvendiren, 2019; Rüdrich et al., 2019). For example, Yilgor *et al.* described the fabrication of 3D printed porous PCL scaffolds with either a basic or crossed fibre orientation, where enhanced MSC osteogenesis was observed in the crossed fibre scaffolds (Yilgor et al., 2008). It has been proposed that substrates exhibiting a high curvature or acute fibre angle augments differentiation and tissue deposition by enhanced cellular tension at those interfaces (Rumpler et al., 2008). For instance, Bidan *et al.* showed a positive correlation between tissue formation and substrates with high curvatures, where osteoblasts deposited extracellular matrix on cross-shaped pores twice as fast when compared to square-shaped pores (Bidan et al., 2013). Hence, the increased substrate curvature of the triangle pore scaffolds may increase cellular tension within osteoblasts and promote their differentiation capacity. We found that cells cultured on scaffolds exhibiting a larger pore size increased osteoblast differentiation (ALP activity, collagen production and calcium deposition) when compared to smaller pores in both conformations, consistent with previous reports (Van Bael et al., 2012; Di Luca et al., 2016). Scaffolds possessing a triangle pore geometry and larger pore sizes exhibited increased porosity and permeability, likely enhancing the cell seeding efficiency of these scaffolds and media diffusivity during culture. Improving scaffold permeability through increasing pore size, has been shown to promote osteoblast differentiation via elevated fluid flow/shear stress (Ouyang et al., 2019). An increasing body of evidence has reported the influence of biomaterial cues as potent regulators of cell fate via altering epigenetic functionality (Li et al., 2011; Tan et al., 2015; Lv et al., 2018a). It is well known that during osteogenic differentiation, the epigenetic landscape of cells is altered. For example, Lee *et al.* showed that HDAC activity is reduced during osteogenesis, which resulted in hyperacetylation induced gene activation (Lee et al., 2006). As such, researchers have harnessed epigenetic modifying compounds including HDAC inhibitors (HDACis) to accelerate osteogenic differentiation both *in vitro* and *in vivo* (Huynh et al., 2016; Man et al., 2021c). Moreover, several studies have demonstrated the role of biomaterials substrates in augmenting key epigenetic functions via the modification of the cells actin cytoskeleton which transmits forces to the nuclear protein lamin A/C, ultimately resulting in enhanced transcriptional activation (Li et al., 2011; Downing et al., 2013). In this present study, our findings showed that osteoblast cultured on triangle pore scaffolds exhibited substantial reduced HDAC activity, resulting in increased histone acetylation levels. The enhanced transcriptional permissiveness induced by hyperacetylation has been reported to accelerate differentiation on biomaterial substrates *via* increased gene activation (Li et al., 2011; Lv et al., 2018b), consistent with the upregulated osteogenic gene expression observed in this study. Thus, these findings indicate the influence of biomechanical cues induced by the different scaffold architectures on augmenting osteogenic differentiation capacity via epigenetic regulation. Therefore, altering the scaffolds architecture through augmenting pore size/geometry has a significant impact in regulating osteoblast differentiation (Abbasi et al., 2020). Moreover, modifying the scaffolds pore geometry and size likely plays a pivotal role in the enhanced regenerative capacity of these constructs used for bone fracture healing.

Due to issues regarding EV diffusion efficiency from conventional scaffold systems and the lack of control in scaffold properties (Patel et al., 2018), 3D printing allows for the reproducible manufacture of constructs with controllable architectural parameters. The present findings show a significant increase in EV production for scaffold-cultured osteoblasts compared to 2D culture, consistent with several studies demonstrating elevated EV yield from 3D model systems (Zhang et al., 2017; Cha et al., 2018). Interestingly, we observed substantial differences in the quantity of EVs isolated from these constructs during osteoblast culture. Scaffolds possessing a triangle pore conformation secreted significantly enhanced EV yield (>2.15-fold) when compared to square pore scaffolds. Moreover, we showed that larger pore sizes resulted in a slight enhancement in EV yield in both pore conformations, although not significant. This EV release profile correlated with the differentiation status of the parental cell, likely impacting osteoblast differentiation and mineralisation observed during scaffold culture via autocrine and paracrine signalling. Interestingly, it has been reported that scaffolds exhibiting an acute pore angle promote osseointegration and *de novo* tissue formation following implantation (Deng et al., 2021). Hence, the influence of the scaffold geometric architecture likely impacts EV yield and potency *in vivo* stimulating fracture healing, although this would require further investigation. Through characterisation of the constructs, we showed that scaffolds with a triangle pore conformation and larger pore sizes exhibited an increased permeability/porosity. The improved scaffold permeability will likely enhance the media diffusivity during culture, impacting both osteoblast differentiation and EV procurement. Several studies have reported the influence of increased fluid flow on osteoblast differentiation, through the activation of mechanotransductive signals (Yourek et al., 2010) and the enhanced bioactivity of secreted EVs (Eichholz et al., 2020b). Hence, augmenting scaffold permeability through architectural modifications could provide a tailorable method to improve EV manufacture. In addition to the influence of scaffold architecture on EV procurement, 3D culture-induced differentiation may also increase EV biogenesis and production through increased intracellular calcium (Savina et al., 2003; Taylor et al., 2020). It has been reported that intracellular calcium is causatively linked to osteogenic differentiation. For example, Wu *et al.* showed that intracellular calcium levels within BMSCs increased during osteogenic differentiation, and the introduction of Thapsingargin, an inhibitor to endoplasmic reticulum calcium ATPase, reduced osteogenesis (Wu et al., 2020). Interestingly, we showed that osteoblasts cultured on triangle pore scaffolds exhibited a significantly elevated intracellular calcium content when compared to cells cultured on other scaffolds designs and 2D. These findings correlated with the quantity of EVs procured during culture. Therefore, EV procurement from these titanium lattices is likely influenced by the scaffold effects on osteoblast differentiation, the cell secretion phenotype and scaffold permeability/porosity.

Several studies have demonstrated the enhanced biological potency of EVs procured from cells cultured in 3D models compared to 2D (Cha et al., 2018; Otto et al., 2021). The use of 3D printing would allow for the fabrication of biomimetic scaffold systems, allowing for the procurement of EVs from cells with a more physiological relevant phenotype when compared to conventional 2D culture. The present results showed that EVs derived from titanium lattices significantly improved hBMSCs osteogenic differentiation when compared to EVs procured from 2D cultured cells. It is well known that culturing cells in more physiological relevant models promotes their osteogenic capacity, thus, it is likely the augmented EV yield and potency induced by modifying the cells substrate, plays a critical role in the enhanced differentiation capacity of cells observed in 3D culture. The data also showed that EVs derived from the triangular pore conformation displayed increased osteoinductive potency when compared to vesicles isolated from cells cultured on the square pore scaffolds. The enhanced osteoinductive efficacy of EVs procured from the triangle pore scaffolds correlated with the differentiation status of the parental cell during osteogenic culture. Increasing evidence has reported the influence of biophysical and biochemical stimulation on augmenting secreted EVs osteoinductive potency. For example, Davies *et al.* reported EVs procured from mineralising osteoblast were significantly enriched in proteins involved in osteoblast communication and extracellular mineralisation such as bridging collagens and annexin calcium channelling proteins when compared to EVs from non-mineralising cells (Davies et al., 2017). Eichholz *et al.* showed that mechanical stimulation of osteocytes accelerated its differentiation and the osteoinductive potency of their EVs via the enrichment of several pro-osteogenic proteins (Eichholz et al., 2020b). Thus, it is likely the accelerated mineralisation induced by the triangle pore scaffolds enriched the secreted EVs with proteins involved in osteoblast differentiation and mineralisation. Additionally, in this study, we reported that osteoblasts cultured on triangle scaffolds elicited substantially altered epigenetic functionality, resulting in increased osteogenic gene activation. Previously we demonstrated that altering the epigenome of mineralising osteoblast via HDACi induced hyperacetylation, significantly enriched secreted EVs with several pro-osteogenic microRNAs and transcriptional regulators which augmented the epigenetic landscape of recipient hBMSCs and accelerated its osteogenic capacity (Man et al., 2021a). Therefore, it is likely the EVs derived from osteoblasts cultured on triangle pore scaffolds were enriched with bioactive factors which modified the epigenetic landscape at recipient hBMSCs increasing its differentiation capacity. Future work elucidating the scaffold-derived EVs mechanisms of actions would be an important area for future studies. Together, these findings indicate the importance of EV parental cell phenotype on the biological potency of their secreted EVs, as these nanoparticles are essentially a fingerprint of their donor cell (Zhang et al., 2019; Sahoo et al., 2021). Moreover, the use of titanium implants exhibiting a triangle pore architecture *in vivo* could facilitate the production of EVs from recruited stem/progenitor cells, further potentiating the scaffolds role in stimulating endogenous bone repair by increasing pro-regenerative secretome signalling. Therefore, the use of AM techniques such as 3D printing allows for the tailorable fabrication of biomimetic culture platforms to enhance the osteoinductive potency of EVs.

In addition to the influence of scaffold architecture on improving material functionality, several studies have investigated the effects of different surface coatings on promoting the biomimetic nature of biomaterials (Kaur and Singh, 2019; Kazimierczak and Przekora, 2020). For example, Eichholz *et al.* developed a bone-mimetic nnHA coating, which enhanced the osteoinductive capacity of PCL scaffolds (Eichholz et al., 2020a). Moreover, it has been reported that the elevation of extracellular calcium levels transiently increases intracellular concentrations through the activation of calcium-sensing receptors (Yanai et al., 2019). Therefore, the application of a calcium-based scaffold coating could promote osteoblast differentiation and EV production. We demonstrated that the application of a nnHA coating significantly enhanced mineralisation of osteoblasts during culture, consistent with several studies in the literature (Habraken et al., 2016; Eichholz et al., 2020a). Interestingly, we observed a significantly increased quantity of EVs procured from the nnHA coated scaffolds (>3.5-fold) when compared to the uncoated groups. This profile correlated with the differentiation status of the parental cell and its secretory phenotype (intracellular calcium levels). The enhanced quantity of EVs released during osteoblast culture on the nnHA coated scaffolds, likely played a pivotal role in promoting the mineralisation observed on the constructs. Interestingly, there was no significant difference in the osteoinductive potency of EVs procured from the nnHA scaffolds compared to the untreated groups. Thus, there is likely a delicate balance between promoting EV yield and the biological potency of these cell-derived nanoparticles. It is important to note, that given the dramatic increase in EV yield induced by nnHA coated scaffolds, the direct implantation of nnHA coated constructs *in situ* would likely promote osteogenesis as a result of enhanced EV production, rather than augmenting the potency at an individual vesicle level. These findings suggest that the application of a nnHA coating would improve the bone formation capacity of titanium substrates, consistent with several studies in the literature (Oonishi et al., 1989; Popkov et al., 2017). Although titanium alloys exhibit a poor degradation profile, the nnHA coating has demonstrated its versatility to improve the osteoinductive potency of non-metallic scaffolds (Eichholz et al., 2020a). Therefore, these findings show that the application of a simple nnHA scaffold coating can significantly augment the production of pro-osteogenic EVs, further enhancing the scalable manufacture of these nanoparticles for clinical use.

In this study, we investigated the influence of 3D printed scaffold architecture on the osteoinductive potency of osteoblast-derived EVs for bone repair. Scaffolds exhibiting a triangle pore conformation and larger pore size displayed increased porosity and permeability. Osteoblast differentiation was significantly accelerated when cultured on triangle pore scaffolds, with increased EV production from these scaffold systems. EV procurement yield correlated with osteogenic phenotype, intracellular calcium levels and scaffold porosity. EVs derived from the triangle pore scaffolds significantly promoted hBMSCs osteogenic differentiation when compared to EVs derived from other scaffold designs. Interestingly, we demonstrated that a simple nnHA coating significantly improved osteoblast osteogenic differentiation and EV yield when compared to the uncoated groups. Future studies would be required to investigate the influence of different scaffold parameters on EV composition, which may be linked to their mechanism of action. A limitation of this study is the lack of *in vivo* assessment which would provide increased pre-evidence into the biological potency of these scaffold-derived EVs in stimulating bone regeneration. Moreover, there is tremendous scope to investigate the influence of combining tailored 3D printed scaffolds with bioreactors systems to further increase the scalable manufacture of therapeutic relevant EVs for clinical applications.

## Conclusion

Together, these findings demonstrate the impact of 3D printed scaffold architecture and surface composition on improving EV manufacture and their therapeutic efficacy, indicating the considerable potential of harnessing bone-mimetic culture platforms to enhance the production of pro-regenerative EVs for bone repair.

## Data Availability

The raw data supporting the conclusions of this article will be made available by the authors, without undue reservation.
