# Supplementary material for: Development of a Bone-Mimetic 3D Printed Ti6Al4V Scaffold to Enhance Osteoblast-Derived Extracellular Vesicles’ Therapeutic Efficacy for Bone Regeneration
Source: Front Bioeng Biotechnol. 2021 Oct 26;9:757220. doi: 10.3389/fbioe.2021.757220 (PMC8576375; doi:10.3389/fbioe.2021.757220)
Supplement: Supplementary file 1 [file DataSheet1.PDF]

Supplementary materials

Supplementary Table 1. Primer sequences used for RT-qPCR

Gene	Forward	Reverse
ALP	ATCGACGTGATCATGGGTGG	TGGGAATGCTTGTGTCTGGG
COL1A1	AGACAGTGATTGAATACAAAACCA	GGAGTTTACAGGAAGCAGACA
OCN	ACCTCACAGATGCCAAGCC-	GCCGGAGTCTGTTCACTACC-

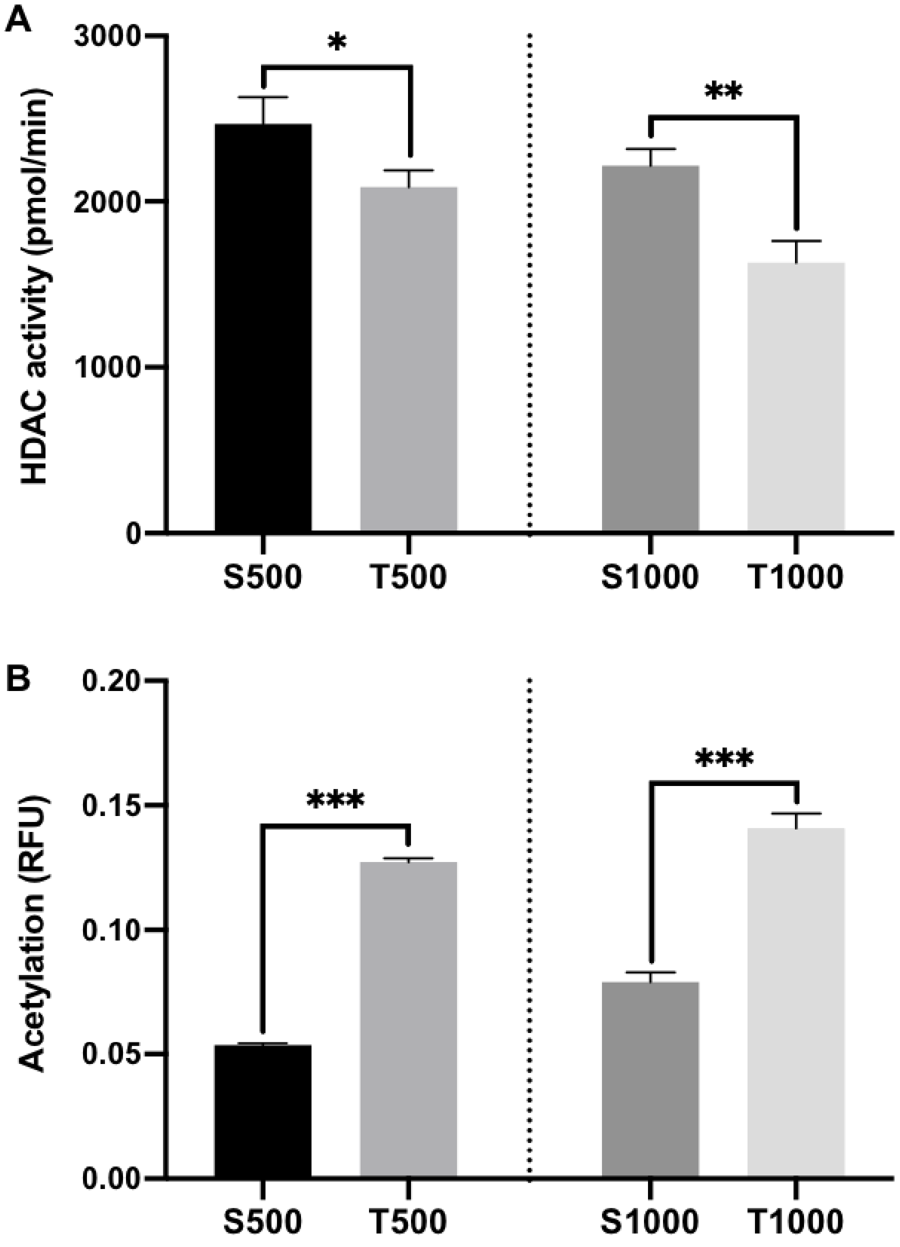

Supplementary figure 1. The effect of titanium scaffold architecture on osteoblast A) HDAC activity and B) histone acetylation. Data are expressed as mean ± SD (n=3). *P ≤ 0.05, **P ≤ 0.01 and ***P ≤ 0.001.

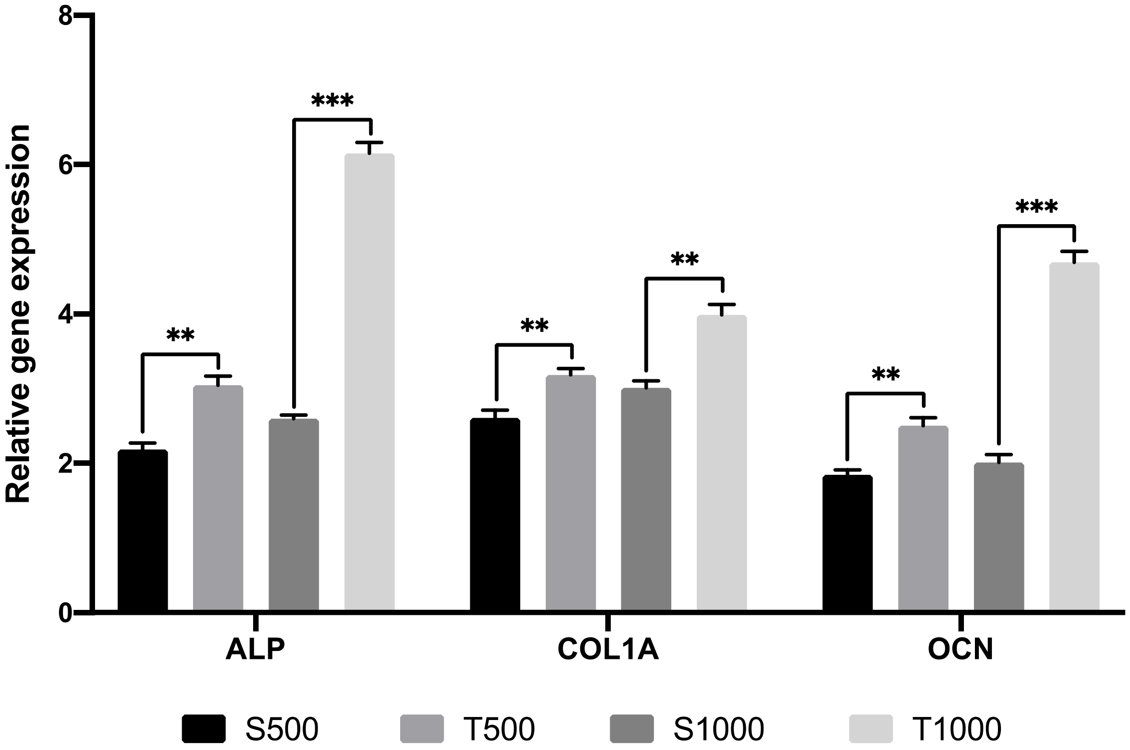

Supplementary figure 2. Gene expression levels of *ALP, COL1A,* and *OCN* from scaffold-cultured osteoblasts during osteogenic culture. Data are expressed as mean ± SD (n=3). **P ≤ 0.01 and ***P ≤ 0.001.

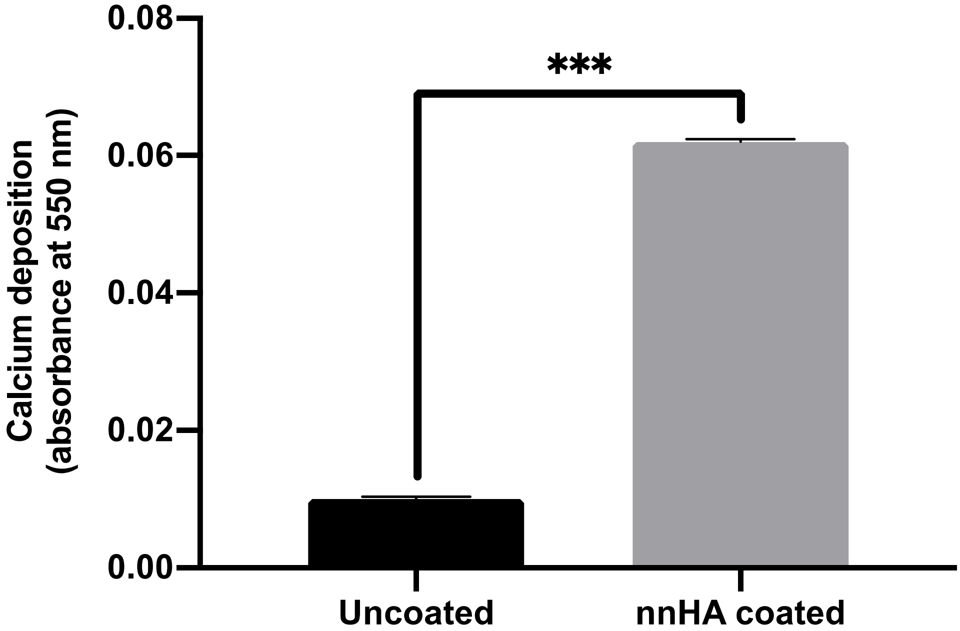

Supplementary figure 3. Quantification of calcium content on nnHA coated scaffolds. Alizarin red staining of uncoated and nnHA coated scaffolds. Data are expressed as mean ± SD (n = 3). ***P ≤ 0.001.
